# Vessel Density Loss of the Deep Peripapillary Area in Glaucoma Suspects and Its Association with Features of the Lamina Cribrosa

**DOI:** 10.3390/jcm10112373

**Published:** 2021-05-28

**Authors:** Soo-Ji Jeon, Hae-Young Lopilly Park, Chan-Kee Park

**Affiliations:** 1 Apgujeong St. Mary’s Eye Center, 859 Eonju-ro, Gangnam-gu, Seoul 06023, Korea; sj8801@gmail.com; 2 Department of Ophthalmology, Seoul St. Mary’s Hospital, College of Medicine, The Catholic University of Korea, 222 Banpo-daero, Seocho-gu, Seoul 06591, Korea; lopilly@catholic.ac.kr

**Keywords:** glaucoma suspect, lamina cribrosa, optical coherence tomography angiography, peripapillary vessel density

## Abstract

Purpose: To investigate the association of decreased vessel density (VD) in the deep peripapillary region and structural features of the lamina cribrosa (LC). Materials and Methods: 70 eyes of glaucoma suspects with enlarged cup-to-disc ratio were scanned and 51 eyes with adequate image quality were included in this study. All subjects had localized VD defects in the deep layer but intact VD in the superficial layer around the peripapillary region using optical coherence tomography angiography (OCTA). Only single-hemizone OCTA results from one eye of each subject had to fulfill the distinctive feature mentioned above to perform inter-eye and inter-hemizone comparisons. The thickness and depth of the LC, and prelaminar thickness were measured using enhanced depth imaging OCT (EDI-OCT). Paired t-tests were performed to evaluate differences in measurements of the LC and prelaminar thickness within each individual. *p*-values lower than 0.05 was considered to be statistically significant. Results: Eyes with deep VD defects in the peripapillary region in OCTA had thinner LC than the fellow eyes. The hemizone with the deep VD defects in the peripapillary region had a thinner LC and a deeper depth of LC than the other hemizone in the same eye. According to logistic regression analysis, a thin LC was a significant factor associated with deep VD defect in the peripapillary region. Conclusions: Glaucoma suspect eyes with deep VD defects in the peripapillary area exhibited structural differences in the LC. The structural changes of the LC was associated with the vessel density in the deep peripapillary layer at the stage of suspected glaucoma.

## 1. Introduction

The pathogenesis of glaucoma has been studied continuously, and a number of hypotheses have been proposed to explain glaucomatous damage of retinal ganglion cells (RGCs). The hypotheses have largely been based on two main theories—structural theory and vascular theory [1,2]. According to structural theory, deformation of the lamina cribrosa (LC) is the presumed site of glaucoma-related neural damage. LC compression and the resultative focal LC defects have been reported to be the important structural changes of glaucoma, and associated visual field (VF) and retinal nerve fiber layer (RNFL) loss were noticeable [3,4]. However, the interest of vascular dysregulation has also been investigated consistently as a factor preceding or coinciding with the onset of glaucoma and its progression [5,6,7,8,9].

Owing to recent advances in optical coherence tomography angiography (OCTA), reproducible and non-invasive methods of vascular status measurement are now widely used [10]. The automated layer segmentation allows for en-face imaging of deep vascular details of the macula and peripapillary area, layer by layer, to choroid [11,12,13].

When analyzing OCTA results by layer, the microvasculature of the superficial retinal layer mainly includes the RNFL, ganglion cell and inner plexiform layer (GCIPL). Glaucomatous damage occurs in the RNFL and ganglion cell layer (GCL); therefore, it is difficult to suggest that decreased vessel density (VD) in the superficial layer could have a causative or resultant relationship with RGC loss. On the other hand, the deep retinal layer including the inner nuclear layer (INL) is relatively less affected by glaucoma, and focusing on changes in vessel density of the deep retinal layer could be useful for monitoring microvasculature changes without the interference of retinal structural thinning in glaucoma patients. The previous studies of our research team have already reported the implications of vessel changes in the deep retinal layer of the macular region [14,15].

In the case of the peripapillary area, we hypothesized that these changes in the deep layer could be meaningful to observe alterations of vessel density without the interference from structural thinning, as occurs in the macula. Sung et al. reported that the peripapillary microvasculature of the deep layer was related to the structural characteristics of optic nerve head (ONH) such as tilt and rotation in myopia [16]. In addition, we detected that there were some patients with localized vessel density defects in the deep layer but intact VD in the superficial layer of peripapillary area among glaucoma suspect subjects in our clinic. These cases raised questions about how deep peripapillary vessel was altered in glaucoma suspect with intact superficial flow.

Therefore, the purpose of this study was to investigate the clinical features to having deep peripapillary VD defects in glaucoma suspect. To minimize the effects of systemic factors on both eyes, we performed inter-eye and intra-eye comparisons.

## 2. Materials and Methods

### 2.1. Study Design and Population

This cross-sectional study was performed according to the tenets of the Declaration of Helsinki and was approved by the Institutional Review and Ethics Boards of Seoul St. Mary’s Hospital, South Korea. The need for written informed consent was waived by our Review Board. We included 70 subjects with a diagnosis of glaucoma suspect from Seoul St. Mary’s Hospital between January 2018 and February 2020.

All subjects underwent comprehensive ophthalmic examinations as in previous OCTA related studies [15]. Examinations included best-corrected visual acuity (BCVA), slit-lamp examination, Goldmann applanation tonometry, gonioscopy, and dilated fundus biomicroscopy. Color fundus and red-free RNFL photographs were obtained with a nonmydriatic retinal camera (Canon, Tokyo, Japan), and standard automated perimetry (SAP) using the Swedish interactive threshold standard algorithm (SITA) 24-2 program (Humphrey Visual Field Analyzer; Carl Zeiss Meditec, Dublin, CA, USA), circumpapillary retinal nerve fiber layer (cpRNFL) and macular GCIPL (mGCIPL) thicknesses were obtained by DRI OCT (Topcon, Tokyo, Japan) for diagnosis of glaucoma suspect. A history of diabetes, hypertension or cerebrovascular disease was documented, and symptoms of hemodynamic instability such as migraine or cold extremities were also recorded. All subjects had open angles on gonioscopy, BCVA of 20/40 or better and intraocular pressure (IOP) of 21 mmHg or lower.

Glaucoma suspect in this study was defined as having normal RNFL thickness in OCT results with only a vertical C/D ratio ≥ 0.5 or asymmetric optic discs in both eyes (asymmetry of C/D ratio between two eyes ≥ 0.2 that was not caused by the difference in optic disc size or shape). Each photograph was reviewed by two independent glaucoma specialists (SJJ and HYP). In case of disagreement of judgement, a senior reviewer (CKP) adjudicated. RNFL thickness was considered to be normal if it was within 95% of the values within the internally embedded database of healthy, age-matched normal population and it was marked in the green color sector of the temporal-superior-nasal-inferior-temporal (TSNIT) graph. Glaucoma hemifield test results of glaucoma suspect were within normal limits based on a minimum of two reliable visual field measures.

Patients were excluded if they met any of the following criteria: (1) a history of non-glaucomatous optic neuropathy; (2) a history of eye trauma or surgery except for uncomplicated cataract extraction; (3) pathologic myopia (chorioretinal atrophy, intrachoroidal cavitation, choroidal neovascularization, lacquer crack) (4) other retinal diseases including diabetic retinopathy and retinal vascular diseases such as vascular occlusion or uveitis.

### 2.2. Identification of Study Subjects with VD Defects in the Deep Peripapillary Area Using OCTA

OCTA images were acquired through DRI OCT Triton system (Topcon, Tokyo, Japan) using a swept source laser with a wavelength of 1050 nm and a raster scan protocol. OCTA is based on Topcon OCT angiography ratio analysis (OCTARA) algorithm that could improve the detection of low blood flow by combining ratio analysis from different scanning intervals [17]. An active eye tracker was used to reduce motion artifact during imaging. In the peripapillary region, automated layer segmentation was performed. For the imaging of superficial peripapillary microvasculature, the radial peripapillary capillary segment extending from the internal limiting membrane (ILM) to the RNFL was analyzed. For the imaging of deep peripapillary microvasculature, the embedded segmentation program demarcated the boundary line from 130 μm below the ILM to 390 μm below the basement membrane including the INL, outer plexiform layer (OPL), outer nuclear layer (ONL), and choroid. Highly myopic eyes with a spherical equivalent < −12.0 D or staphyloma were excluded for clear image acquisition around the ONH. The images with image quality scores over 70 were selected.

**Figure 1:**
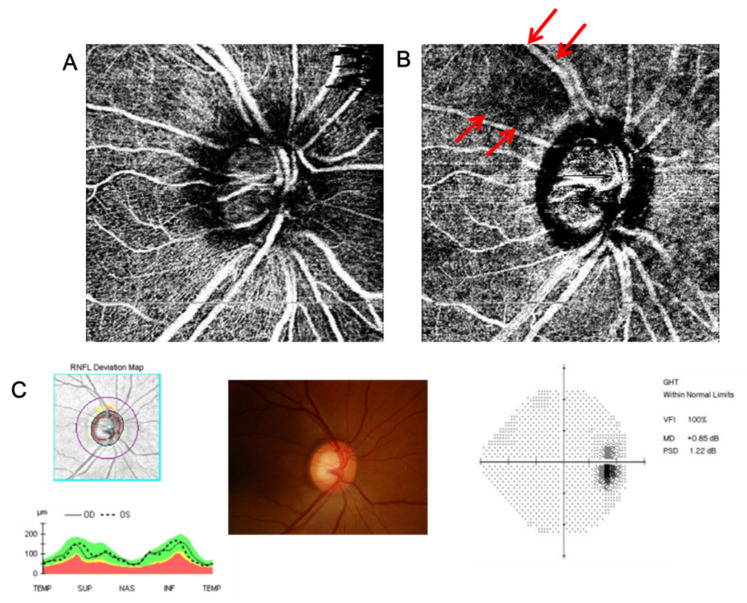
(**A**) Superficial peripapillary OCT angiography in the superficial layer (from the retinal nerve fiber layer (RNFL) to 130 μm below the internal limiting membrane (ILM) including the ganglion cell-inner plexiform (GCIPL) layer). (**B**) Deep peripapillary OCT angiography in the deep layer (from 130 μm below the ILM to the basement membrane including the inner nuclear layer (INL)). (**C**) RNFL OCT and visual field test with increased cup-disc ratio (CDR) which indicate glaucoma suspect. There are subjects among glaucoma suspect patients with wedge-shaped VD defect in the deep layer of peripapillary area (indicated as arrows in **B**), even though the vessel density in the superficial layer is unaffected (intact vascular status in **A**).

From OCTA images, we identified the study subjects with VD defects in the deep layer of peripapillary area but with intact superficial VD. Figure 1 represents the subjects meeting these criteria. Systemic vascular factors could function as a confounding factor in assessing the relationship between peripapillary vessel density and the structural change of LC. Therefore, in order to minimize the influence of the systemic factors, we included only subjects with decreased deep peripapillary VD in one eye and normal VD in the opposite eye to perform inter-eye comparison in one subject and inter-hemizone comparison in one eye.

### 2.3. Lamina Cribrosa Measurements Using Enhanced Depth Imaging OCT

Lamina cribrosa imaging was performed using enhanced depth imaging OCT (EDI-OCT) device (Heidelberg Engineering, Heidelberg, Germany). Radial 2D b-scans centered on the optic disc were obtained with the Heidelberg Spectralis OCT (Spectralis software version 5.1.1.0, Eye Explorer Software 1.6.1.0) using a wavelength of 870 nm providing up to 40,000 A scans/sec, with a depth resolution of 7 μm and a transversal resolution of 14 μm.

The detailed measurement of the lamina cribrosa and prelaminar thickness using EDI-OCT was performed as described previously [18,19]. The thickness and depth of the LC were measured at three different locations; mid-superior, central, and mid-inferior. The LC thickness was defined as the distance between the anterior and posterior borders of the highly reflective region at the vertical center of the ONH in the horizontal cross-sectional B-scans. For LC depth and prelaminar thickness measurements, we drew a reference line connecting both ends of Bruch’s membrane opening. A perpendicular line was drawn from the center of the reference line to the anterior border of LC in each B-scan, which we defined as LC depth. Similarly, the prelaminar thickness was measured along the perpendicular line from the anterior border of the reflective region to the anterior border of the highly reflective region, which is the anterior laminar border.

For inter-eye comparisons, the mean of the three measurements (mid-superior, central, and mid-inferior) was defined as the LC thickness, LC depth, and prelaminar thickness. For inter-hemizone comparisons within each eye, three locations of the same interval from each hemizone were measured and the mean of these three measurements was also used as the representative value.

Two independent observers (SJJ and HYP) each repeated LC measurements, three times as mentioned above. The values of six results were averaged, and the mean value was used in the analyses. To assess interobserver and intraobserver reproducibility, intraclass correlation coefficient (ICC) was calculated from 15 randomly selected measurements. The interobserver ICC for LC thickness was 0.915, ICC for LC depth was 0.927, and ICC for prelaminar thickness was 0.894. In addition, intraobserver ICC showed excellent agreement of the LC and prelaminar thickness—ICC was 0.975, 0.988 and 0.971 for LC thickness, LC depth and prelaminar thickness, respectively.

Only images with quality scores > 20 were used, and when necessary, the images were re-examined. Each EDI scan included an average of 20 OCT frames; if more than three of the radial scans were unrecognizable, the eye was excluded. We excluded patients with lens opacity that could interfere image quality and localization of lower LC border.

### 2.4. VF Sensitivity and Macular Vessel Density

VF sensitivity was calculated as the mean value of the threshold in the pattern deviation map of SITA 24-2. Measurement of macular vessel density in OCTA using binary slab images were performed as described in previous studies [14,15,20]. In short, the binarized images created using ImageJ software (National Institutes of Health, Bethesda, MD, USA) were segmented into an area of interest and background after using the “adjust threshold” tool, which automatically sets the lower and upper threshold values. After the white pixels were designated as vessels and the black pixels as background, the vessel density was calculated as a percentage of the total area of the white pixels divided by the total pixel area of the image.

## 3. Statistical Analysis

All data are presented as means ± standard deviation. The interobserver and intraobserver agreements for LC measurements and prelaminar thickness were assessed by calculation of intraclass correlation coefficients (ICC). Paired t-tests were used for inter-eye and inter-hemizone comparisons of continuous variables. Chi-square tests were used to compare categorical variables between eyes. For logistic regression analyses, variables with *p* values < 0.3 in univariate analyses were included in the multivariate analysis. *p* value threshold of 0.1 could include only one variable in the multivariate analysis, we designated *p* value threshold of 0.3 to include multiple variables. All statistical analyses were performed with SPSS version 24.0 (IBM Corp., Armonk, NY, USA). *p* < 0.05 was considered to be statistically significant.

## 4. Results

This study initially included 70 subjects with a diagnosis of glaucoma suspect. Among them, there were 19 subjects with poor OCTA or EDI-OCT images that were subsequently excluded from our analyses. Table 1 described the baseline characteristics of study subjects. The number of eyes with a superior location of deep VD defects with our interest in OCTA was greater than those with an inferior location.

Table 2 showed the inter-eye comparisons of study subjects. The eyes with VD defects in the deep peripapillary area had a thinner LC thickness than the fellow eyes with intact VD (*p* < 0.001). However, the cpRNFL thickness, mean deviation (MD) and mean sensitivity of VF were not statistically different.

Based on Table 3, we performed inter-hemizone comparisons between each subject’s hemizone with deep VD defect and the unaffected other. The hemizone with deep VD defect had worse VF sensitivity (*p* < 0.001). In addition, the LC thickness was thinner and the LC depth was deeper in the hemizone with deep VD defect (*p* < 0.001 and *p* = 0.014, respectively). In other words, the LC of the hemizone with deep VD defect was thinner and more deeply located than the hemizone without deep VD defect.

**Figure 2:**
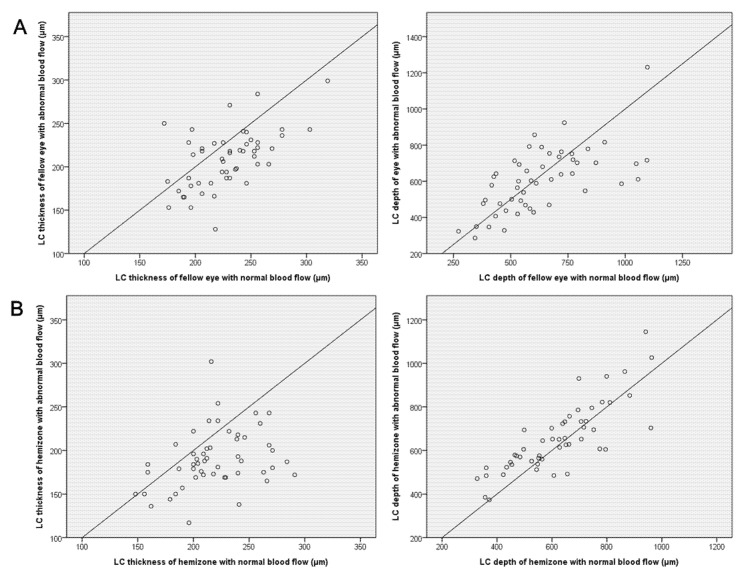
Scatter plots showing comparisons of the thickness and depth of lamina cribrosa (**A**) between eyes with deep vessel density (VD) defects and the contralateral eyes with intact VD, and (**B**) between hemizones with deep VD defects and the other hemizones with intact VD in OCT angiography.

The inter-eye or inter-hemizone correlations between LC thickness and depth are described in Figure 2 using scatterplots. Most eyes with deep peripapillary VD defects had thinner lamina. Most hemizones with deep peripapillary VD defects also had thinner and even deeper LC.

The factors associated with presence of deep peripapillary VD defect were evaluated using logistic regression analyses (Table 4). In univariate and multivariate analyses, the LC thickness was a significant factor associated with deep VD defect in the peripapillary area. The eyes with thinner LC had higher probability of showing deep peripapillary VD defect.

**Figure 3:**
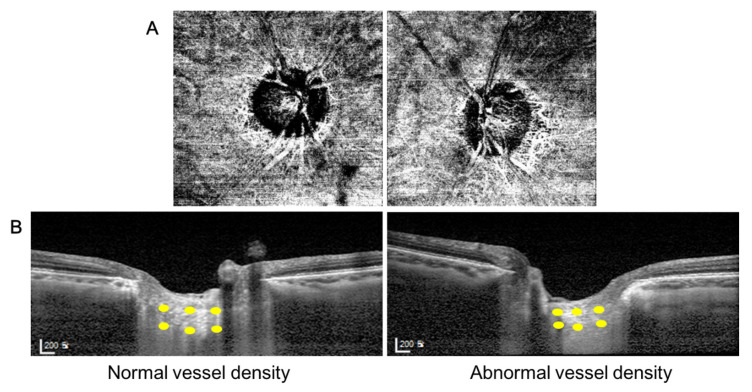
A representative case. (**A**) A 60-year old woman with decreased deep OCTA vessel density of peripapillary area in her left eye. (**B**) The thickness of lamina cribrosa in her left eye with abnormal vessel density was thinner than that in her right eye with normal vessel density.

**Figure 4:**
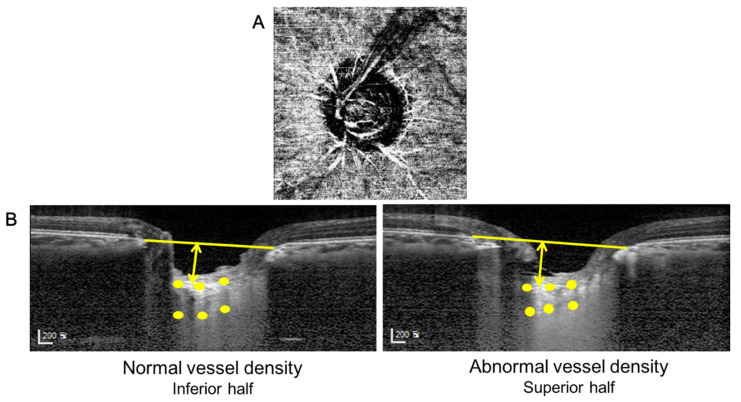
A representative case. (**A**) A 47-year old man with deep VD defect in his superotemporal peripapillary area of left eye. (**B**) The lamina cribrosa of his superior half was thinner and more deeply located than that of his inferior half.

Representative cases are presented in Figure 3 and Figure 4. The eyes of a 60-year-old woman with deep peripapillary VD defect in her left eye showed thinner LC compared with her unaffected right eye (Figure 3). In Figure 4, the left eye of a 47-year-old man showed deep peripapillary VD defect in the superotemporal region. The left EDI-OCT image was from the inferior half with an intact OCTA and the right image was from the superior half with deep VD defect. The LC thickness was thinner and LC depth was deeper in his superior hemizone than the inferior.

## 5. Discussion

The present study started with the identification of altered vessel density from the deep peripapillary area including INL, OPL, ONL and choroid among glaucoma suspects. In these cases, the superficial peripapillary vessel density in the RNFL and GCL was intact with normal range of structural thickness of the layer. We tried to search for the distinctive clinical characteristics of those eyes compared to glaucoma suspect eyes without deep peripapillary vessel density alteration. Both structural and functional traits were investigated by comparing bilateral eyes of one subject. The reason we conducted the inter-eye and inter-hemizone comparisons was to minimize the effect of systemic conditions on the vessel density of the ONH.

Tepelus et al. previously suggested the decreased choroidal vascular parameters within the macula and ONH area in glaucoma patients compared with normal controls [21]. The OCTA changes of choriocapillaries located in the deep peripepillary area was called as microvascular dropout and have been focused in glaucoma [22,23,24]. Likewise, several studies have consistently asserted there should be a focus on the changes occurring in vessels in the deep layer of microvasculature in glaucoma [25,26,27]. Sung et al. reported that the deep peripapillary microvascular reduction was associated with structural characteristics of the optic disc, such as tilt in myopic eyes [16]. The differential mechanical strain near the ONH could affect the structure of the optic disc and peripapillary microvasculature, and consequently, might affect the eye’s susceptibility to glaucoma development.

In the present study, the eyes with deep peripapillary VD defects had thinner LC than the fellow eye. In addition, the hemizone with abnormal deep peripapillary OCTA images showed not only thinner but had a more deeply located LC than the other hemizone. LC thickness was the factor associated with peripapillary VD defects in the deep layer. This finding was consistent with previous reports that structural characteristics of the optic disc were related with microvascular changes in the peripapillary area. To our knowledge, this is the first study investigating microvasculature alterations in glaucoma suspect to consider the changes occurring in the deep peripapillary layer as from the INL to the choroid.

While the mechanism explaining the association between changes in the LC and microvasculature change might not be definite, we have several hypotheses. First, eyes with thin LC may intrinsically have weak structure against mechanical strain on the optic disc. Structural weakness may be consistent in the extracellular matrix (ECM) of microvasculature. The weak ECM would interfere with capillary integrity [28] and, in accordance, could result in increased microvascular vulnerability to vascular dysregulation. Second, it is also possible that the retinal artery within the deep retina (INL, OPL and ONL) could be physically obliterated when the LC itself becomes compressed as a result of increased strain around the ONH. According to Prada et al. [29] the characteristics of the microvasculature around the ONH in the laminar and prelaminar region could affect the blood flow of the deep retina. In other words, strain over the ONH could subsequently effect the small microvasculature of the deep retina piercing the LC and flowing to the deep layer of peripapillary area. Since the vessels of the superficial retina are larger than those of the deep retina [30], it might be possible that only the microvascular change in the deep retina appeared before changes to the superficial retina could occur under the normal range of IOP with gentle strain. From our results, though not statistically significant, the deep macular vessel density of the hemizone with VD defect trended toward decrease compared with the other hemizone (42.71% vs. 45.29%, *p* = 0.147).

Our results also showed that the hemizone with deep VD defect had worse VF mean sensitivity than the unaffected other. Shin et al. previously reported that vessel density was associated with corresponding VF sensitivity [31]. From the study of Arend et al., altitudinal VF asymmetric defects were associated with the different circulation of retina in glaucoma patients [32]. According to Chen et al., reduced microcirculation was found even in the normal hemisphere of glaucoma patients [5]. These studies may partially support our finding of decreased VF sensitivity accompanying microvasculature changes. In the future, after several associated further studies, the deep VD changes could be used as a surrogate implying visual function alterations in glaucoma patients.

This study had several limitations. First, as a cross-sectional study, it was not possible to determine whether a causal relationship existed between structural changes of the LC and alterations in peripapillary vessel density. In the future, a longitudinal study including large populations with various cause and effect possibilities should be performed. Second, since we selected the well performed OCTA images of the deep peripapillary layer, it was mainly the eyes of the subjects who cooperated best with the data acquisition that were included in the analyses; these were mainly young and myopic eyes without lens opacity. It is necessary to study eyes with various age groups and axial lengths to ensure the generalized application of study results. Third, disc tilt could affect the measurements of lamina cribrosa. To minimize the possibility of image distortion, we used b-scan images of 1:1 μm for real measurements. Fourth, subjects with systemic disease such as diabetes or hypertension could have preclinical vascular alteration despite excluding patients with apparent retinopathy to minimize effect of systemic disease. In the future, studies with large populations, and the association between systemic disease and deep peripapillary vascular circulation should be studied. Finally, in this study, we restricted the subjects to those with glaucoma suspect for minimizing the effect of structural thinning of the retina on vascular obliteration. Nevertheless, the VF sensitivity of hemizone showed difference based on the presence of decreased vessel density in the deep peripapillary area. Though the results of this observational study might be a long way from clinical application, if the possibility of glaucoma development from glaucoma suspect is investigated through longitudinal study based on the results of this study, it might be possible to find a novel way of clinical application of OCTA on glaucoma development.

In conclusion, there were eyes with the diagnosis of glaucoma suspect exhibiting VD defect only in the deep layer of peripapillary area and not in the superficial layer. The regions with altered vessel density had different LC structure compared with the other regions. In other words, altered LC structure was associated with decreased vessel density in the deep peripapillary area in glaucoma suspect. Even at the stage of glaucoma suspect with intact RNFL, pre-clinical changes of vessel density accompanied with LC change were detected. Though the causal context between LC structure and vessel density change in the peripapillary area could not be demonstrated in this cross-sectional study, we speculated that the different pattern of lamina structure could affect the vascular status near the optic nerve head. The cause and effect relationship between LC structure and deep vessel density changes around the ONH should be investigated in future study. Further, the progression toward glaucoma from glaucoma suspect with VD defects, or, continuous VD defects change in glaucoma suspect should be studied using longitudinal study to identify the possibility of clinical application of deep VD defects.

## Figures and Tables

**Table 1:** Comparisons of baseline characteristics of study subjects (*n* = 51).

Age (years)	49.92 (±13.85)
Axial length (mm)	25.13 (±1.60)
Male:Female	21:30
Hypertension (%)	9 (17.64)
Diabetes (%)	4 (7.84)
Systemic vascular dysregulation (%) *	9 (17.64)
Right eye with deep VD defects (%)	14 (27.45)
Superior location of deep VD defects (%)	32 (62.74)

* Systemic vascular dysregulation included history of migraine, cold extremities, cerebrovascular problems, angina or arrhythmia.

**Table 2:** Comparisons between eyes with deep VD defects and fellow eyes without deep VD defects.

	Eyes with Deep VD Defects (*n* = 51)	Fellow Eyes without Deep VD Defects (*n* = 51)	*p* Value
Disc hemorrhage (%)	3 (5.88)	2 (3.92)	0.647
BCVA (decimal)	0.96 (±0.08)	0.93 (±0.13)	0.245
Intraocular pressure (mmHg)	16.69 (±4.48)	16.43 (±3.89)	0.760
Axial length (mm)	25.10 (±1.55)	25.17 (±1.66)	0.485
RNFL OCT variables			
cpRNFL thickness	89.25 (±8.73)	89.73 (±9.06)	0.790
Rim area	1.12 (±0.27)	1.07 (±0.22)	0.268
Disc area	2.17 (±0.51)	2.07 (±0.45)	0.309
Average C/D ratio	0.66 (±0.12)	0.67 (±0.11)	0.859
Vertical C/D ratio	0.63 (±0.12)	0.63 (±0.11)	0.986
Cup vlume	0.42 (±0.27)	0.41 (±0.25)	0.804
SITA 24-2 MD (dB)	−0.68 (±1.46)	−0.51 (±1.52)	0.569
SITA 24-2 mean sensitivity (dB)	29.72 (±1.72)	30.29 (±1.82)	0.299
LC thickness (μm)	208.92 (±33.56)	228.57 (±31.93)	<0.001
LC depth (μm)	607.71 (±175.26)	631.51 (±202.67)	0.297
Prelaminar thickness (μm)	109.44 (±41.96)	113.82 (±54.45)	0.521
Macular VD			
Superficial layer (%)	36.05 (±2.24)	35.82 (±2.40)	0.660
Deep layer (%) *	43.47 (±3.25)	43.47 (±2.84)	0.608

Paired *t*-test was used. VD: vessel density; LC: lamina cribrosa * Macular vessel density of deep layer mainly included the area of inner nuclear layer.

**Table 3:** Inter-hemizone comparisons in eyes with deep VD defects.

	Hemizone with Deep VD Defects	Hemizone without Deep VD Defects	*p* Value
cpRNFL thickness			
ISNT map *	112.82 (±13.73)	111.64 (±18.65)	0.771
Clock-hour map †	93.22 (±13.77)	88.71 (±17.78)	0.176
SITA 24-2 mean sensitivity (dB)	29.40 (±1.99)	30.17 (±1.83)	<0.001
LC thickness (μm)	189.82 (±33.50)	219.16 (±34.46)	<0.001
LC depth (μm)	657.37 (±158.86)	622.63 (±162.02)	0.014
Prelaminar thickness (μm)	146.31 (±83.86)	162.33 (±81.51)	0.296
Macular vessel density of deep layer (%) ‡	42.71 (±4.96)	45.29 (±7.06)	0.147

Paired *t*-test was used. VD: vessel density; LC: lamina cribrosa. * Superior and inferior RNFL thickness were compared. † Superotemporal (12, 1, 2 o-clock) and inferotemporal (4, 5, 6 o-clock) RNFL thickness were compared. ‡ Macular vessel density of deep layer mainly included the area of inner nuclear layer.

**Table 4:** Factors associated with deep VD defects from OCTA.

	Univariate			Multivariate		
	Exp(ß)	95% CI	*p* Value	Exp(ß)	95% CI	*p* Value
Disc hemorrhage	1.531	0.245 to 9.574	0.649			
Axial length	1.020	0.774 to 1.344	0.887			
SITA 24-2 MD (dB)	0.924	0.706 to 1.209	0.565			
SITA 24-2 sensitivity (dB)	0.825	0.573 to 1.188	0.288	0.864	0.600 to 1.244	0.432
RNFL OCT variables						
cpRNFL thickness	0.994	0.951 to 1.039	0.788			
Rim area	2.540	0.481 to 13.429	0.272	2.094	0.124 to 34.798	0.606
Disc area	1.528	0.677 to 3.451	0.297	1.110	0.199 to 6.208	0.905
Average C/D ratio	0.733	0.025 to 21.331	0.857			
Vertical C/D ratio	0.970	0.032 to 29.428	0.986			
Cup volume	1.214	0.268 to 5.513	0.801			
LC thickness (μm)	0.980	0.967 to 0.993	0.003	0.980	0.962 to 0.999	0.041
LC depth (μm)	0.999	0.997 to 1.001	0.524			
Prelaminar thickness (μm)	0.998	0.990 to 1.006	0.651			
Macular VD (%)						
Superficial layer	1.043	0.866 to 1.257	0.656			
Deep layer	1.000	0.868 to 1.153	0.997			

Variables with *p* values < 0.3 on univariate analysis were included in the multivariate analysis. *p* value threshold of 0.3 was designated to include multiple variables in the multivariate analysis. VD: vessel density; OCTA: optical coherence tomography angiography; MD: mean deviation; LC: lamina cribrosa.
